# Slip Correction Measurements of Certified PSL Nanoparticles Using a Nanometer Differential Mobility Analyzer (Nano-DMA) for Knudsen Number From 0.5 to 83

**DOI:** 10.6028/jres.110.005

**Published:** 2005-02-01

**Authors:** Jung Hyeun Kim, George W. Mulholland, Scott R. Kukuck, David Y. H. Pui

**Affiliations:** Department of Mechanical Engineering, Particle Technology Lab, University of Minnesota, 111 Church Street, Minneapolis, MN 55455; National Institute of Standards and Technology, Gaithersburg, MD 20899; Department of Mechanical Engineering, Particle Technology Lab, University of Minnesota, 111 Church Street, Minneapolis, MN 55455

**Keywords:** electrospray, Knudsen number, NMDA, polystyrene latex particles, slip correction factor

## Abstract

The slip correction factor has been investigated at reduced pressures and high Knudsen number using polystyrene latex (PSL) particles. Nano-differential mobility analyzers (NDMA) were used in determining the slip correction factor by measuring the electrical mobility of 100.7 nm, 269 nm, and 19.90 nm particles as a function of pressure. The aerosol was generated via electrospray to avoid multiplets for the 19.90 nm particles and to reduce the contaminant residue on the particle surface. System pressure was varied down to 8.27 kPa, enabling slip correction measurements for Knudsen numbers as large as 83. A condensation particle counter was modified for low pressure application. The slip correction factor obtained for the three particle sizes is fitted well by the equation: *C* = 1 + *Kn* (*α* + *β* exp(−*γ*/*K*n)), with *α* = 1.165, *β* = 0.483, and *γ* = 0.997. The first quantitative uncertainty analysis for slip correction measurements was carried out. The expanded relative uncertainty (95 % confidence interval) in measuring slip correction factor was about 2 % for the 100.7 nm SRM particles, about 3 % for the 19.90 nm PSL particles, and about 2.5 % for the 269 nm SRM particles. The major sources of uncertainty are the diameter of particles, the geometric constant associated with NDMA, and the voltage.

## 1. Introduction

Stokes’s law is a solution for the drag force (*F*_d_) of a rigid sphere obtained by solving the Navier-Stokes equations in the viscous limit of Reynolds number ≪ 1. The solution imposes no-slip at the particle surface and, therefore, assumes that the relative velocity of the fluid is zero at the surface. This assumption begins to break down for particle diameters several times the gas mean free path when such particles experience “slip” at their surface. One manifestation of this effect is that such particles settle more rapidly than predicted by Stokes’s law. By including a slip correction factor *C*, Stokes law can be modified to apply for particle diameters on the order of the gas mean free path and smaller:

$$F_{D}=-\frac{3\pi \mu \nu D_{p}}{C(Kn)}$$ (1)

where,

*Kn* = Knudsen number (2/*D*_p_)
*D*_p_ = particle diameter
*λ* = mean free path in the liquid or gaseous phase
*µ* = gas viscosity
*ν* = particle velocity relative to the fluid.

The negative sign indicates that the drag force act opposite the direction of the particle velocity. While there are analytic expressions for the slip correction in the limit of particle size large compared to the mean free path (Stokes) and small compared to the mean free path (Epstein), there have not been quantitative calculations for the intermediate region.

Many studies have been carried out to characterize the slip correction factor as a function of Knudsen number. In 1910, Cunningham [1] derived a correction factor, (1 + *A* · *Kn*) including a positive parameter *A*, for the Stokes drag force required to maintain the fluid velocity in the high Knudsen number regime. The Cunningham factor always reduces the Stokes drag force. Using the Cunningham correction factor, application of Stokes law can be extended to the particle sizes comparable to or less than the mean free path of the gas molecules. Later, several experimental investigations were performed to obtain empirical equations of the slip correction factor for a wide range of Knudsen numbers. In the same year with Cunningham, Millikan [2] experimentally verified the linear dependence of the correction term on mean free path in Cunningham’s formula for the Knudsen number less than 0.3. Knudsen and Weber [3] expressed the parameter *A* as a function of Knudsen number in a form consistent with experiments at larger Knudsen numbers:

C(Kn)=1+A⋅Kn (2)

A=α+β⋅exp(−γ/Kn) (3)

where *α*, *β*, and *γ* are experimentally determined constants. They determined the constant values from the damping of torsional oscillation of a pair of glass spheres suspended in a vessel at reduced pressures.

After Knudsen and Weber’s results reported the new form of the slip correction factor, a number of experimental studies were performed to determine *α*, *β*, and *γ* for the parameter *A*. From 1910 to 1923, Millikan and his students measured the constants for various particle surfaces and gas media. In 1923, Millikan [4] used his classic oil drop method to determine value of the parameter *A* for a wide range of values of Knudsen number: from 0.5 to 134 with the mean free path of 94.17 nm in air. The values were found to agree with the predicted low and high Knudsen number limits to within the experimental relative uncertainty of ±2 %. In his work, oil drops of size ranging from 2.6 µm to 245 nm were observed at pressures ranging from 101.3 kPa down to 0.2 kPa.

After Millikan’s result, the constants used to determine the parameter A were modified to account for a more accurate representation of the mean free path by several authors: Langmuir [5], Davies [6], DeMarcus and Thomas [7], Reif [8], and Fuchs [9]. A summary of these studies and the resulting mean free paths is reported by Allen and Raabe [10] who fully re-evaluated the Millikan oil drop results by least square fitting the data using a mean free path of 67.3 nm at 23 ºC and 101.3 kPa. Three years later, Allen and Raabe [11] reported the slip correction factor measured for micrometer size polystyrene spheres using an improved version of the Millikan apparatus. Their measurements covered a Knudsen number range from 0.03 to 7.2.

Rader [12] re-analyzed the slip correction factor for small particles in nine common gases in 1990. He reviewed the oil-drop work of Ishida [13] in the continuum slip regime (*Kn* ≤ 0.4) and provided accurate values for the parameter for nine gases: air, argon, helium, hydrogen, methane, ethane, isobutene, nitrous oxide, and carbon dioxide. He then used the oil-drop works of Eglin [14] and Millikan [4] for slip correction data over a wide range of Knudsen number (0.2 to ≈95) in air, oxygen, carbon dioxide, and helium to determine the values in the parameter *A*. Five years later, Hutchins et al., [15] used modulated dynamic light scattering to find the slip correction factor for solid (polystyrene and polyvinyltoluene) spherical particles of diameter ranging from 1.00 µm to 2.12 µm. They measured the drag forces on spherical particles suspended in dry air using dynamic light scattering measurements to determine the diffusion coefficient of a single levitated particle from which the slip correction factor could be obtained.

The constants *α*, *β*, and *γ* in previous slip correction parameters of the Knudsen and Weber form are summarized in Table 1. It is important when comparing the values to use the same mean free path for air at standard conditions. We have used a mean free path of 67.3 nm at a standard temperature of 23 ºC and a standard pressure of 101.3 kPa for the results shown in Table 1.

In this study, we determine the slip correction by measuring the electrical mobility of the particles as a function of pressure. The mobility distributions of the nominally monodisperse aerosols are measured for each pressure condition using a condensation nucleus counter to detect the particles. This method has the advantage of measuring the mobility of about 10^4^ particles during one minute of sampling. In most previous studies of slip correction, single particles were used with one study typically reporting on measurements of, at most, a hundred particles or so. Another advantage of mobility analysis is the ability to measure the slip correction for smaller particle sizes, down to a nominal diameter of 3 nm, as the method does not require direct observation of the particles to make a measurement. Many applications of aerosol science are for atmospheric conditions where it is important to know the validity of the slip correction function as a function of decreasing particle size at a fixed pressure. The 20 nm, 100 nm, and 270 nm diameter particles studied here span much of the Knudsen number region of interest.

To enhance the accuracy of the results, two of the three particle samples studied were NIST Standard Reference Materials (SRM^®^ 1691, 269 nm; SRM^®^ 1963, 100.7 nm). The third particle sample was a nominal 20 nm particle size, accurately sized using a Nano-Differential Mobility Analyzer (NDMA).

## 2. Theory

The electrical mobility, *Z*_p_, of a singly charged particle can be determined by equating the electric field force and the Stokes drag force,

$$Z_{p}=\frac{e\cdot C}{3\pi \mu D_{p}}$$ (4)

where *e* is the electron charge. From Eq. (4), the slip correction factor *C* can be rewritten as:

$$C=\frac{Z_{p}\cdot 3\pi \mu D_{p}}{e}$$ (5)

Knutson and Whitby [16] obtained an expression for the electrical mobility of particles in a differential mobility analyzer (DMA) of cylindrical geometry by matching the time of particle movements between the radial direction, from the outer cylinder of aerosol inlet to the inner cylinder of aerosol exit, under a certain electric field and the vertical direction, from the sheath air inlet to outlet. It is expressed as:

$$Z_{p}=\frac{Q}{2\pi VL}\ln(r_{2}/r_{1})$$ (6)

where

*Q* = sheath flow rate
*V* = center rod voltage
*r*_1_ = inner radius of NDMA
*r*_2_ = outer radius of NDMA
*L* = characteristic length of NDMA.

It is convenient to express the combination of geometric factors as a single geometric constant, *G*_f_, defined as

$$G_{f}=\frac{\ln(r_{2}/r_{1})}{2\pi L}.$$ (7)

Substituting the expression for *Z*_p_ intoEq. (5), the following expression for the slip correction factor is obtained:

$$C=\frac{Q\cdot G_{f}\cdot (3\pi \mu D_{p})}{V\cdot e}.$$ (8)

With the use of a DMA originally developed by Knutson and Whitby [16], the size resolution is proportional to the aerosol/sheath flow ratio in the classifying region. The resolution of the DMA is reduced for a low aerosol/sheath flow ratio because of the mismatch of aerosol and sheath flow velocities at the wide inlet slit of the DMA due to the flow recirculation as shown by Chen et al. [17]. Chen et al. suggested a new inlet design to reduce the recirculation problem and then used a similar approach in the inlet design of the NDMA [18]. Here, the slit width is reduced to improve flow velocity matching in the classifying region and to avoid electric field penetration into the upstream side of the entrance slit. As a result, the NDMA has the potential for high resolution and low uncertainty in sizing and classifying nanosize particles.

## 3. Fluid Properties

Before describing the experimental approach, we first present the key fluid properties for computing the slip correction factor, the viscosity and the mean free path.

### 3.1 Viscosity of Air

Millikan [4] used an air viscosity of *µ*_23_ = 1.824 × 10^−5^ kg m^−1^ s^−1^ for his slip correction experiments from an average of the most accurate measurements taken in the early 1900s. In 1945, Birge [19] reported the weighted average value of the viscosity of air, *µ*_23_ = (1.83245 ± 0.00069) × 10^−5^ kg m^−1^ s^−1^ from six different results, correcting for temperature by using the Sutherland equation. This air viscosity value was used in recent studies by Allen and Raabe [10,11] and by Hutchins et al. [15]. For consistency, we also consider the Birge result as the reference viscosity for this study. Once the reference viscosity at 23 °C is determined, the viscosity for other temperatures can be obtained using the Sutherland formula as discussed by Allen and Raabe [10],

$$\mu_{T}=\mu_{23}{\left(\frac{T}{T_{0}}\right)}^{1.5}\left(\frac{T_{0}+110.4}{T+110.4}\right)$$ (9)

where *T*_0_ is the absolute reference temperature (296.15 K) and *T* is the absolute temperature. The viscosity of gas approaches a definite limit (the low-density limit) as the pressure approaches zero at a given temperature; for most gases including air, the limit is reached at 101.3 kPa [20].

### 3.2 Mean Free Path of Air

The mean free path of air, *λ*, cannot be directly measured, but instead is determined from the kinetic theory relationship for viscosity,

$$\mu =\phi \rho \bar{c}\lambda$$ (10)

where *ø* is a constant dependent upon the intermolecular potential, *ρ* is the gas density, and $\bar{c}$ is the mean velocity of gas molecules. Table 2 shows a summary for different kinetic models in determining mean free path. Millikan [4] used *ø* = 0.3502 to determine a value for the mean free path of 94.17 nm at 101.3 kPa and 23 °C. The reason for such a large difference in *ø* lies in the persistence of molecules moving in their original direction after a collision. More detailed discussion about this can be found elsewhere [21]. The latest researchers (Allen and Raabe [10,11] and Hutchins et al. [15]) used *λ*_0_ = 67.3 nm with *ø* = 0.491 for the mean free path of air at 101.3 kPa and 23 °C in determining their slip correction factor experimentally. This value for *ø* is derived by assuming hard elastic spheres with repulsive forces between the molecules and is, therefore, not exact for a diatomic nitrogen molecule. For consistency with previous work, we use this value, *ø* = 0.491, with the caution that others using our results for the slip correction parameter must use the same definition of the mean free path when computing the Knudsen number. This choice also allows us to compare our results with the previous studies. Once the reference value of *λ*_0_ has been chosen, it can be corrected for any pressure and temperature with Willeke’s relation [22]

$$\lambda =\lambda_{0}\left(\frac{T}{T_{0}}\right)\left(\frac{P_{0}}{P}\right)\left(\frac{1+110.4/T_{0}}{1+110.4/T}\right)$$ (11)

where,

*λ*_0_ = 67.3 nm, for air at *T*_0_, *P*_0_
*T*_0_ = reference temperature, 296.15 K
*P*_0_ = reference pressure, 101.3 kPa
*T* = air temperature inside the classifier, K
*P* = air pressure inside the classifier, kPa.

The values of the mean free path and the viscosity of air used in estimating the slip correction factor are summarized in Table 3 along with the value of the electronic charge.

## 4. Experimental Method

The aerosol system for measuring the slip correction factor consists of an electrospray particle generation unit, a NDMA unit for determining the particle mobility, and a modified condensation particle counter to monitor the particle concentration. The individual components are described below.

### 4.1 Monodisperse Generation System

#### 4.1.1 Electrospray

**Fig. 1:**
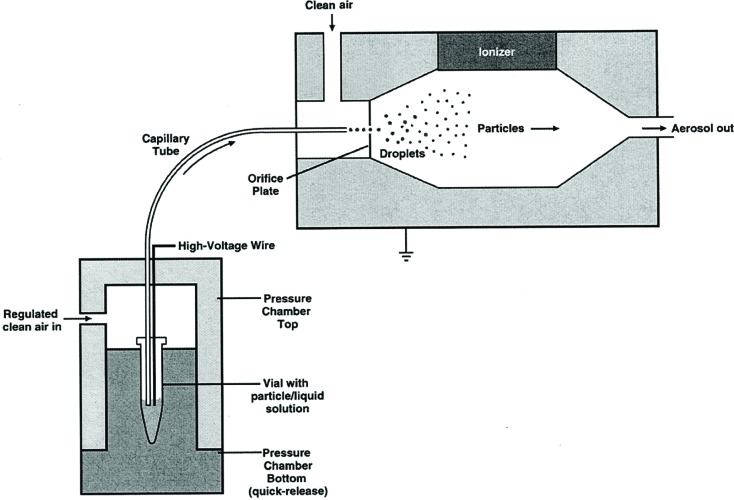
Schematic diagram of the electrospray adapted from the TSI 3480 technical manual.

The electrospray system used to generate polystyrene aerosol particles from a water suspension is shown in Fig. 1. The sample solution is stored in a vial that is enclosed in a cylindrical pressure chamber. The chamber accommodates a capillary and a platinum high-voltage wire, both of which are immersed in the solution. Differential pressure causes the solution to be pushed through the capillary. A voltage control regulates the electric field exerted at the capillary exit that draws the charged solution out of the capillary and forms droplets that are mixed with clean filtered and dehumidified air. The mixed sheath flow transports the aerosolized droplets to a chamber where the highly charged droplets are brought to a Boltzmann equilibrium charge distribution using a Polonium 210 source. The liquid droplets, claimed to have a nominal size of 200 nm by the manufacturer, quickly evaporate before entering the classifier, leaving individual PSL particles and nominal 8 nm residue particles that result from the evaporation of droplets not containing a PSL particle. Even though the expected size of the droplets is smaller than the 270 nm PSL particles, electrospraying was still able to aerosoloize 270 nm PSL particles. In this study, a 40 µm inner diameter capillary was used with a capillary pressure drop of 25.6 kPa for a liquid flow rate of 1.1 × 10^−6^ cm^3^/s (66.0 nL/min). Information on the theory and use of electrospray is reported by Chen et al. [24].

Standard reference materials with known sizes of 100.7 nm and 269 nm were diluted for electrospraying. In the following we shall use the nominal sizes of 100 nm and 270 nm when referring to these SRM particle sizes. One drop of the 100 nm size and a half drop of the 270 nm size from the original bottles having a mass concentration of approximately 0.5 % were added to a 1 cm^3^ vial of 20 mol/m^3^ ammonium acetate buffer solution with a conductivity of 0.2 S/m (Siemens = 1/Ω). The applied voltage to the plate electrode was adjusted until the electrospray produced a stable cone-jet. If the electrospray was unstable from capillary clogging, measurements were stopped and the capillary replaced or unclogged. Clogging was a serious problem in the generation of the 270 nm PSL particles. In order to prevent clogging problem, the solution of the 270 nm particles was further diluted four times with buffer solution. The typical particle density for doubly charged particles was about 1 cm^−3^. In addition to the two SRM particles, 20 nm particles from a commercial vendor were also electrosprayed. One drop of the particle suspension was diluted with the 1 cm^3^ vial buffer solution. The mean size and uncertainty of the particles is summarized in Table 4.

#### 4.1.2 Particle Size Calibration for 20 nm

There are no accurately sized, monodisperse particle standards available at 20 nm. Our approach is to use particles with a nominal 20 nm diameter from a commercial source and then use a NDMA to select a given particle size. By rearrangingEq. (8) as shown below, one can determine the particle diameter from the same type of measurements as for determining the slip correction factor *C*,

$$D_{p}=\frac{C(Kn)Ve}{3\pi \mu QG_{f}}$$ (12)

In this case the slip correction factor, *C*(*Kn*), must be known to compute the particle diameter. We use the slip correction parameter, *A*(*Kn*), determined by a best fit to the data for the 100 nm particles in Sec. 6.2 [Eq. (33)] to compute *C*(*Kn*). The *Kn* number for a 100 nm particles at reduced pressure is equivalent to that of the 20 nm particles at atmospheric pressure. The procedure for finding the best fit to the data is presented in Sec. 6.2. Also,Eq. (12) is an implicit equation for *D*_p_, because the quantity *C* is a function of diameter. The equation is solved iteratively for *D*_p_ with a result of 19.90 nm.

For the 20 nm particles, then, a second NDMA was introduced between the electrospray and the second NDMA used to measure the slip correction factor. This unit was always operated at nominal atmospheric pressure and with fixed voltage. For identification purposes, this NDMA is referred to as the sizing NDMA. The aerosol outlet was then introduced into the second NDMA for slip correction measurements at atmospheric and reduced pressures. The second NDMA (or the sole device for measurements on standard particles) is referred to as the measurement NDMA.

### 4.2 Operation of the NDMA at Low Pressure

**Fig. 2:**
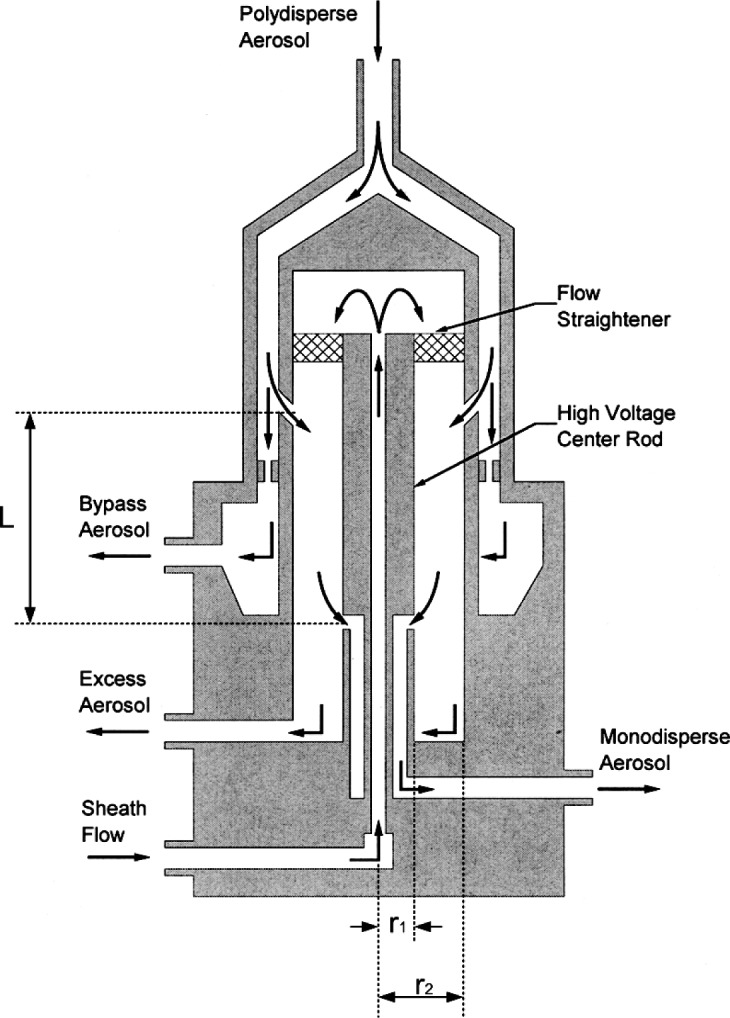
Schematic diagram of an NDMA adapted from the TSI 3085 technical manual.

A schematic of the NDMA system, TSI 3085^1^, is shown in Fig. 2. Briefly, the NDMA has a center electrode outer radius of 0.937 cm and a grounded electrode with an inner radius of 1.905 cm. In order to reduce the effects of diffusion, the characteristic length has been reduced to 4.987 cm from 44.369 cm of the long DMA. The characteristic length is defined as the length between middle of inlet slit to middle of outlet slit as shown in Fig. 2. The aerosol flows through a short connecting tube that quickly widens in a conical section to reach a narrow annular channel. This design promotes axisymmetric aerosol flow and reduces distortions of the flow field. To accommodate the axial aerosol inlet, the sheath air flow is routed through the center electrode from the bottom through the Dacron screen flow straightener while the outer cylinder carries the monodisperse sample flow from the exit slit to the exit port. In this study, the bypass aerosol hole was closed.

**Fig. 3:**
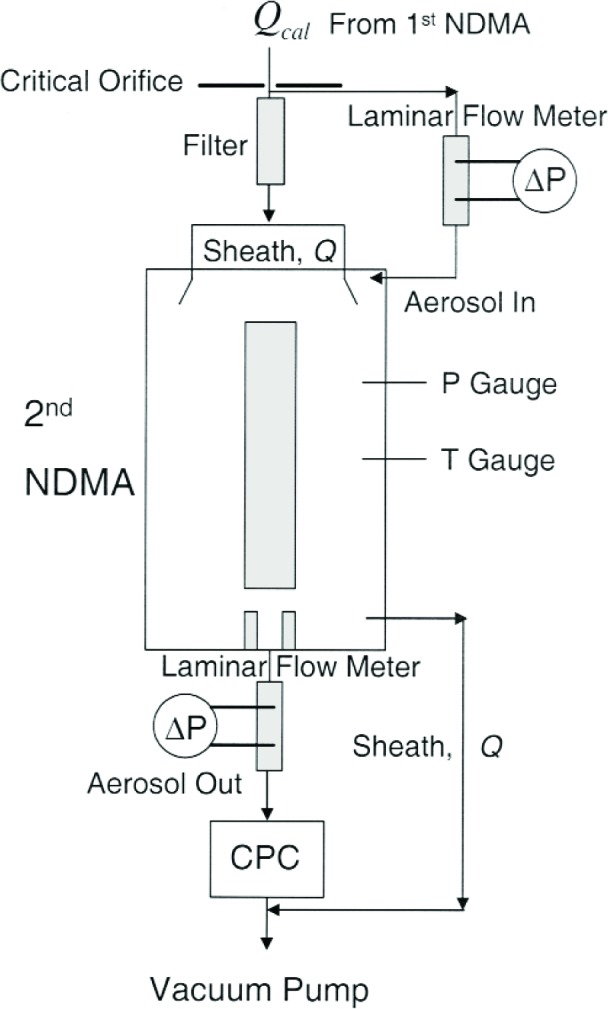
Schematic diagram of the measurement NDMA system for reduced pressure conditions.

The experimental challenge was to introduce a known aerosol and sheath flow into the NDMA at reduced pressures as low as 8.27 kPa without leaks and with minimum flow uncertainty. As shown in Fig. 3, an orifice tube was used for the inlet to the NDMA with the aerosol pressure reduced to the desired level using a vacuum pump. The volumetric flow calibration made use of an accurately calibrated flow meter upstream of the orifice, operating at near ambient pressure. The corresponding volumetric flow for the reduced pressure was obtained using the ideal gas equation of state:

$$Q=Q_{\text{cal}}\cdot \frac{T}{T_{\text{cal}}}\cdot \frac{P_{\text{cal}}}{P}$$ (13)

where,

*Q*_cal_ = volumetric flow rate at *T*_cal_ and *P*_cal_
*Q*_s_ = volumetric flow rate after expansion at reduced pressure
*T*_cal_ = calibration temperature
*P*_cal_ = calibration pressure
*T* = actual temperature at the sheath air inlet
*P* = actual pressure inside the second NDMA.

Temperature measurements were made just before the orifice and at the inlet to the NDMA to monitor any temperature change at the orifice from the rapid expansion of the gas. Temperature was equilibrated to the ambient value as it flowed through the tubing, filter, and laminar flow elements before entering the NDMA. The expanded flow was divided into sheath flow *Q* and aerosol flow, controlled with two laminar flow meters. In this configuration, if both inlet and outlet aerosol flow rates are matched, the sheath air flow rate is determined by subtracting the aerosol flow rate from the total expanded flow rate. During a set of peak voltage measurements, the sheath flow variation was less than 0.2 % based on readings of the differential pressure of the laminar flow meters. The aerosol particles exiting from the measurement NDMA go directly into the condensation particle counter. Both the sheath air and the particle counter outlets were connected to a Leybold Trivac ARS 40–65 vacuum pump with a control valve for achieving a desired pressure level. During measurements, system temperature, differential pressure of the laminar flow meters, and absolute pressure were carefully monitored for further corrections.

Leaks in the plumbing after the orifice either external or internal to the NDMA seriously affect the quality of the data. The leak rate based on sealing the orifice inlet, the aerosol outlet, and the sheath outlet was 0.13 kPa per 3 min at 4.0 kPa system pressure, so the estimated leak is about 1.6 × 10^−3^ cm^3^/s for the approximate system volume of 25 cm^3^. This leak flow is about 0.002 % of the total flow. Initially we observed a much higher leak rate of 0.25 cm^3^/s. By tightening all o-ring junctions inside NDMA and by sealing all Swagelok junctions using vacuum grease, we were able to reduce the leak rate.

### 4.3 Measurement of Experimental Variables

#### 4.3.1 Volumetric Flow Rate

The inlet flow rate to the measurement NDMA was measured using a Drycal DC-Lite flow meter calibrated by a laboratory standard primary piston prover. For reduced pressure conditions,Eq. (13) was used to obtain a flow rate based on the measured volumetric flow rate at experimental conditions.

#### 4.3.2 Temperature

Temperature measurements were made using two ultra-stable probe thermistors, type CSP Thermoprobes manufactured by Thermometrics Inc., with a standard uncertainty of 0.01 °C over the range of 0 °C to 50 °C. The thermistors were installed at the inlet of sheath air and at the inlet of aerosol flow for the measurement NDMA.

#### 4.3.3 Pressure

The pressure measurement system included an MKS Baratron type 690A absolute pressure transducers and a MKS 270D high accuracy signal conditioner. The range of the pressure transducer is from 133 Pa to 133 kPa, and the response time constant is less than 40 ms. The aerosol flow rate through a laminar flow meter at reduced pressure was monitored using a MKS Baratron type 398HD differential pressure gauge (Max:13.3 kPa) together with a second MKS 270D digital readout.

#### 4.3.4 Voltage

A power supply (Bertan, model 205B-10R) was used for the center rod voltage of the NDMA after calibration with a resistive voltage divider and a standard digital voltmeter, which is used at NIST for calibrating DMA voltages over the range of 10 000 V to 10 V. The divider is designed for use with a high impedance digital voltmeters with an accuracy of about 0.05 %. All measured voltages were corrected based on the calibration data. This correction is important for the slip correction measurements in the high Knudsen number regime because the measured voltages are small. For example, the peak voltage of 100 nm particles was 8470 V at 98.8 kPa (Kn = 1.4) and 572 V at 5.07 kPa (*Kn* = 27) for the same flow condition. If there were a 5 V deviation from the correct voltage, the effect on particle mobility would be 0.06 % at *Kn* = 1.4 and about 1.0 % at *Kn* = 27. This voltage error would, in turn, result in a corresponding error in the slip correction factor.

#### 4.3.5 Particle Counter

A condensation particle counter (CPC), TSI model 3010, was used to measure particle number concentration as the NDMA voltage was changed. Experimentally the key quantity was the peak voltage. The system had aerosol flow rates ranging from 0.2 L/min to 1.5 L/min depending on the level of system pressure. As the system pressure was reduced, the particle counting efficiency decreased because of the reduced vapor condensation on the PSL sphere and because of diffusional particle loss to the tube wall after the critical orifice. Fortunately, we could measure the number distribution of the PSL spheres for pressures as low as 5.07 kPa to determine the location of peak voltage. Below 5.07 kPa, it was difficult to find the peak voltage with the condensation particle counter because of the low counting capability. The typical peak particle concentration at 5.07 kPa was 0.03 cm^−3^. In measuring number concentrations, one minute of sampling time was used for each datum point to obtain an average value. For the one minute sampling time, the concentration of particles ranged from 1100 cm^−3^ for 20 nm particles at 41.2 kPa to 0.03 cm^−3^ for 100 nm particles at 5.07 kPa.

The purge air flow, usually used for clean room applications, was sealed off to obtain a lower limit of system pressure and more stable pressure during experiments. Vacuum grease was used for the connections at the CPC inlet and outlet to minimize the leakage. In addition, the critical orifice originally installed in the TSI 3010 for the constant flow of 1 L/min was removed from the back side of the laser block because the aerosol flow rate varied depending on the system pressure. A CPC has been used previously in the upper troposphere [25] at pressures as low as 16.0 kPa. Before reducing the system pressure, the condensing fluid (butanol) was removed from the liquid reservoir to prevent flooding. An adequate amount of fluid remained in the wick for growing droplets large enough to be detected by the counter.

#### 4.3.6 Geometric Constant *G*_f_

The geometric constant *G*_f_ is computed based on the cylindrical NDMA dimensions: *r*_1_ = 0.937 cm, *r*_2_ = 1.905 cm, and *L* = 4.987 cm (See Fig. 2). The corresponding value of *G*_f_ is 2.264 m^−1^. The tolerances given by the manufacturer are 0.0006 cm for *r*_1_, 0.0013 cm for *r*_2_, and 0.032 cm for *L*.

### 4.4 Measurements of Peak Voltage

**Fig. 4:**
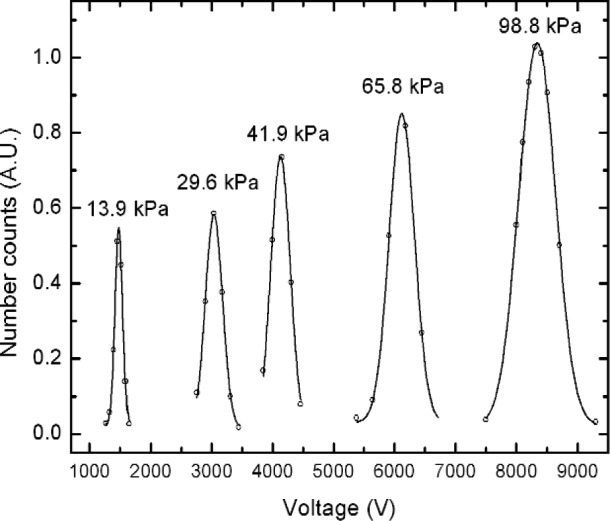
Peak voltage shift at reduced pressure conditions for the 100 nm, SRM 1963 particles. The peak locations were adjusted to a sheath air flow condition of 100 cm^3^s and the peak number concentrations were expressed in arbitrary units (A.U.) to fit on the graph.

Examples of the measurements for peak voltage at reduced pressures using SRM 1963 are shown in Fig. 4. All data points were taken with 1 minute of averaging time for the number concentration. Peak voltages were calculated by Gaussian fit for all sets of data. At reduced pressure conditions, the actual sheath flow is obtained after setting the aerosol flow with laminar flow meters and the associated pressure gauge. Laminar flow meters were used for aerosol flow rates up to 2 L/min. Sheath flow rates were varied from 2.2 L/min to 15.5 L/min depending on the experimental pressure levels. For comparison purposes, it is convenient to consider adjusted peak voltages based on equal volumetric flows through the classifier. The adjustment factor that multiplies the experimental peak voltage, here, is the ratio of the measured sheath flow to 6 L/min. As shown in Fig. 4, the peak locations move to a lower voltage region as the system pressure decreases. This is due to the increase in mean free path with a decrease in system pressure, seeEq. (11), which increases the slip phenomenon.

An extensive series of peak voltage measurements were carried out for the 100 nm particles at ambient pressure conditions to assess the repeatability. As shown in Table 5, the repeatability for the peak voltage was about 5 V for a peak value of about 8300 V.

**Fig. 5:**
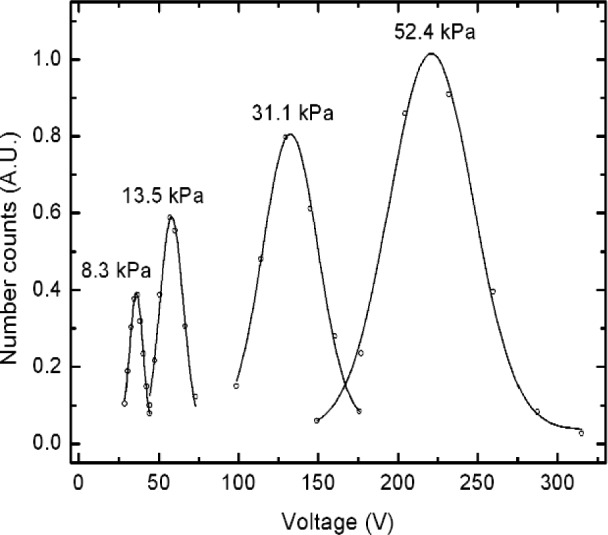
Peak voltage shift at reduced pressure conditions for the 20 nm PSL particles calibrated using SRM 1963. The peak locations were adjusted to a sheath air flow condition of 100 cm^3^/s.

As the pressure of the gas decreases, the electrical breakdown voltage will also decrease. The breakdown voltage sets the limit for the minimum mobility that can be measured at a given pressure. The breakdown voltage was measured for the NDMA as a function of pressure with results shown in Table 6. The table shows that the measured peak voltages of the 100 nm particles are always lower than the breakdown voltage of air for the same pressures. The peak voltage measurements for the 20 nm and the doubly charged 270 nm particles were also less than the breakdown voltage. Figure 5 shows the peak voltage locations for the 20 nm particles at reduced pressure.

## 5. Results

The slip correction factor was calculated usingEq. (8) based on the measured sheath flow rate and peak voltage. Measurement results for the slip correction factor *C* for 100 nm particles are listed in Table 7a for various system pressures, along with values of the slip correction parameter *A*. Similar results for the 270 nm particles are listed in Table 7b for atmospheric and reduced pressure conditions. The relatively large 40 V difference between the two measurements for the atmospheric case is a result of the low sheath flow, low particle concentration, and the short measurement time caused by clogging of the electrospray capillary. Table 7c shows the results of the slip correction factor *C* and the slip correction parameter *A* measured with 20 nm PSL particles. For the 20 nm particles, the size was determined, using the results of the 100 nm measurements, before the values of C and *A* were computed. The procedure for doing this was described in Sec. 4.1.2.

## 6. Uncertainty Analysis for the Slip Correction Parameter

This section presents the uncertainty analysis for the slip correction parameter *A*. There are two parts to the uncertainty analysis. One part is the estimation of Type A uncertainty, uncertainty that is evaluated utilizing statistical methods. In our case the statistical method involves analysis of the residuals, which are the differences between the data points and a nonlinear best fit. Such an analysis has not been carried out previously for the slip correction and is presented in Sec. 6.2.

The other part of the uncertainty analysis is the determination of the Type B uncertainty. Type B uncertainties, in our case, include; manufacturers’ specifications, such as the tolerances for the geometric dimensions of the NDMA; calibration data, including the uncertainties in the PSL SRMs; and scientific judgment. We present the Type B analysis first in Sec. 6.1.

The total combined Type A and Type B uncertainties are computed and results presented in Sec. 6.3. Also, the expanded uncertainty is computed and confidence intervals are obtained at the 67 % and 95 % confidence level for both the slip correction parameter *A* and the slip correction factor *C*.

### 6.1 Type B Uncertainty Analysis

Type B uncertainties are those uncertainties in a measurement obtained by other than statistical means. These uncertainties are generally, but not exclusively, attributed to systematic uncertainties or unknown biases in the components required to obtained the measured result. Consider, for example, the above mentioned uncertainty in the SRMs and in particular consider the uncertainty in the diameter of these particles. The calculations for both the slip correction factor and the Knudsen number require knowing what the diameters of these particles are. We only know, however, what the true diameters are to within some confidence interval. If the true diameter of the 100.7 nm particles were in actuality 110.0 nm, this would propagate through the calculations, altering the results. The goal of the Type B analysis is to quantify the possible deviations that may arise from these factors.

The computation of the Type B uncertainty in *A* requires combining, in an appropriate manner, the Type B uncertainties of the components used in the computation This is done by using what is commonly referred to as the “law of propagation of uncertainty.” This expression can be derived, for a general measured quantity *y*, by considering the differential of the expression used to calculate *y*,

$$dy={\sum_{i}\frac{\partial y}{\partial x_{i}}|}_{x_{i}}dx_{i},$$ (14)

which, to first order, approximates the deviation of the measured quantity due to deviations in the variables, d*x_i_*. If the quantities d*x_i_* are independent random variables, then the variance of d*y* can be expressed as,

$$\sigma_{dy}^{2}=\sum_{i}{\left({\frac{\partial y}{\partial x_{i}}|}_{x_{i}}\right)}^{2}\sigma_{{dx}_{i}}^{2}.$$ (15)

The variances of the independent variables are estimated from information on hand. The uncertainty in *y* then, which estimates a standard deviation, is expressed as,

$$u(y)={\left[\sum_{i}{\left(\frac{\partial y}{\partial x_{i}}\right)}^{2}{\left[u(x_{i})\right]}^{1/2}\right]}^{1/2}$$ (16)

where *u* (*x_i_*) is the standard uncertainty in *x_i_*, an estimate of the standard deviation. It should be remembered that this analysis assumes that the *x_i_* variables are independent.

A simpler form of the above expression, which will be used often in the following, can be obtained if *y* has the following functional form:

$$y=x_{1}^{a}x_{2}^{b}\ldots .$$ (17)

UsingEq. (16), the relative combined standard uncertainty, *u*_r_ (*y*) = *u* (*y*)/*y*, is found to be:

$$u_{r}(y)=u(y)/y={\left[a^{2}{\left(\frac{u_{x_{1}}}{x_{1}}\right)}^{2}+b^{2}{\left(\frac{u_{x_{2}}}{x_{2}}\right)}^{2}+\ldots \right]}^{1/2}$$ (18)

Both Eqs. (16) and (18) will be utilized in the following analysis.

A caveat to the above discussion is that in some cases, such as the determination of the peak voltage, a specific functional form is not available to be used in equation (16). In this case, the relative standard uncertainties are computed utilizing a basic root-sum-of-squares method:

$$u_{r}(y)=u(y)/y={\left[{\left(\frac{u_{x_{1}}}{x_{1}}\right)}^{2}+{\left(\frac{u_{x_{2}}}{x_{2}}\right)}^{2}+\ldots \right]}^{1/2}.$$ (19)

Before proceeding with discussions about the uncertainties in the quantities used to measure the slip correction parameter, we make a statement regarding the determination of component uncertainties. What are required in Eqs. (16, 18–19) are estimates of standard deviations (standard uncertainties). Often, however, the available information does not provide a direct estimate. One example of this is the tolerances provided for the geometric dimensions of the NDMA. Information from the manufacturer stated that the tolerances, ± *δ*, represent the greatest deviations possible from the given dimension. The standard uncertainty for these quantities is, therefore, estimated from this information by assuming an equal probability for the dimension to be anywhere in the tolerance interval, i.e., a rectangular probability distribution. For such a distribution, one finds by integration that the standard deviation (estimated standard uncertainty) is equal to $\delta /\sqrt{3}$.

While previous studies [10, 11, and 15] have enumerated Type B uncertainties, they have not indicated whether they are 1 sigma, 2 sigma, or tolerances. They also have not given an explicit expression for the combined uncertainty. We begin the following discussion with estimates for the Type B uncertainties in the individual quantities used to calculate the slip correction parameter. We then proceed to computing the Type B uncertainty for *A*.

#### 6.1.1 Particle Diameter

The standard relative uncertainty in the number mean diameter of the SRM 1963 particles (100.7 nm) is 0.5 % as reported by Mulholland et al. [26,27]. The SRM 1691 particles (269 nm) have a 0.68 % uncertainty in the number mean diameter. As will be seen below, the standard relative uncertainty for the 19.90 nm particles is 0.63 %.

#### 6.1.2 Pressure

**Fig. 6:**
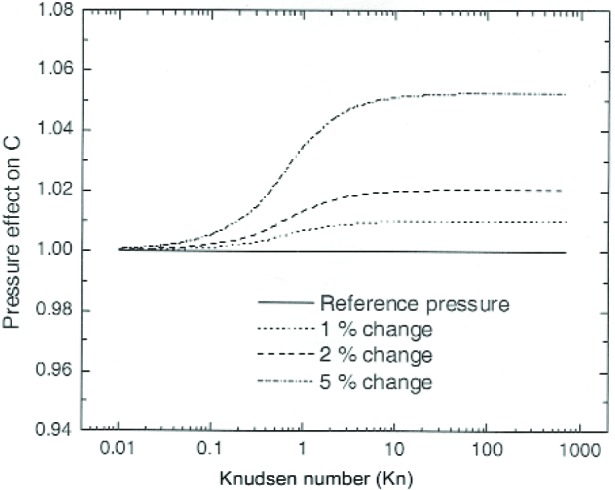
Effect of pressure uncertainty on the measurement of the slip correction factor.

Pressure uncertainty affects the measurement of the slip correction factor both from the flow measurement, equation (13), and from the mean free path, equation (11). A 1 % change in the pressure can produce a 1 % change in *C*(*Kn*) at high *Kn* as observed in Fig. 6. In this case it is the pressure effect on the mean free path of the gas that produces the change. The standard uncertainties of the absolute and differential pressure gauges used in this study are known as 0.12 % from the vendor, and we consider this as the standard relative uncertainty for pressure measurement.

#### 6.1.3 Temperature

The standard uncertainty in the two thermisters is 0.01 °C. However, there is a slight drift in the temperature during a voltage scan and there is also a slight difference in the temperature at the measurement point to the temperature in the NDMA. The estimated standard uncertainty associated with both of these effects is 0.1 °C. The corresponding relative standard uncertainty is 0.03 %. This represents a negligible contribution (less than 1 % of the combined uncertainty) when added in quadrature with the major terms, which are at least 0.5 %. Thus, the temperature uncertainty is neglected in the following uncertainty analyzes.

#### 6.1.4 Flow Rate

The standard relative uncertainty in the meter used to measure flow is 0.06 % from device calibration. At reduced pressure conditions, the total flow is then calculated usingEq. (13) and is, therefore, a function of the measured pressure and temperature and the calibration pressure and temperature. We neglect the temperature uncertainties, see above, but must include both of the pressure measurements, *P* and *P*_cal_. The relative uncertainty in the total flow is calculated usingEq. (16), and is 0.18 %. The sheath flow used in the calculation is the total flow less the aerosol flow. There is also, then, a 0.2 % flow uncertainty in aerosol flow from reading of the differential pressure gauge. However, this effect is negligible, less than 0.5 %, because the aerosol flow is typically only 10 % of the sheath flow.

#### 6.1.5 Peak Voltage

The uncertainty in the peak voltage has three components: that arising from the discrete digital readout from the meter, that arising from its calibration, and that arising from the ability to locate the peak. The discretization uncertainty for the voltmeter is 0.5 V. The corresponding value of the relative uncertainty is estimated as 0.05 % for the 100 nm measurement case and 0.5 % for the 20 nm cases because the peaks are located at different magnitudes for the voltage, i.e., 1000 V for the 100 nm particles and 100 V for the 20 nm particles. The standard relative uncertainty in voltage due to calibration, which is carried out using an accurate 10 000 to 1 divider circuit and digital voltmeter, is estimated as 0.2 %. The standard uncertainty associated with locating the peak was determined to be 0.06 % for the 100 nm and 269 nm particles and 0.15 % for the 20 nm particles by comparing peak values obtained using both Gaussian and Lorentzian fits. Without a direct functional form, we combine the three sources of uncertainty in quadrature usingEq. (19). The resulting standard relative uncertainty is 0.21 % for the SRM particles and 0.56 % for the 20 nm particles.

#### 6.1.6 Set Voltage

In the case of the nominal 20 nm diameter particles, the voltage of the first NDMA, which determines the particle size, is set at a fixed voltage of 398 V. The discretization reduced uncertainty in this case is 0.5/398 = 0.13 %. The only other source of uncertainty is the 0.2 % from voltage calibration. Combining these two terms in quadrature gives a result for the relative voltage uncertainty of 0.24 %. This value is used in estimating the uncertainty in the 20 nm particle size.

#### 6.1.7 Viscosity

The value of the viscosity of air at 23 °C from Birge [19], has 0.04 % relative standard uncertainty. The nominal 7 % relative humidity of the aerosol flowing through the NDMA results in an estimated 0.08 % standard relative uncertainty in the air viscosity. Computing the standard relative uncertainty of viscosity using equation (19), a value of 0.09 % is obtained.

#### 6.1.8 Geometric Constant *G*_f_

The tolerances for the dimensions for the NDMA are given in Sec. 4.3.6 as 0.0006 cm for *r*_1_, 0.0013 cm for *r*_2_, and 0.032 cm for *L*. The standard uncertainty is obtained from the tolerance by dividing by $\sqrt{3}$ as explained in Sec. 6.1. The resulting relative standard uncertainties are 0.04 % for *r*_1_ and *r*_2_ and 0.37 % for *L*. The uncertainty in *G*_f_, defined byEq. (7) is computed using the law of propagation of uncertainty,Eq. (16) based on the relative uncertainties of the quantities *r*_1_, *r*_2_, and *L*. The resulting relative standard uncertainty is 0.38 %.

#### 6.1.9 Settling Distance ∆*L*

Gravitational settling of the particles, motion beyond that of the gaseous flow, can introduce error into the electrical mobility computed usingEq. (4). Settling distance was examined for the lowest pressure case, as it has the largest effect on the slip correction factor. The gravitational settling distance for the 100 nm particles at 50.8 kPa and 22.5 °C was 2.76 × 10^−6^ m for the experimental precipitation time (0.2 s) of the NDMA. The effect is, therefore, only about 0.005 % on the characteristic length of 4.987 cm. The settling length is also negligible for the 270 nm particles. For the effect to be considered significant, i.e., at least 0.04 % of the length *L*, the particle diameter must be increased to 0.5 µm.

#### 6.1.10 Mean Free Path

The mean free path of the gas, *λ*, is a function of pressure and temperature, although the uncertainty in temperature is neglected. The reference conditions are assumed to posses no uncertainty, but rather are by definition. We further neglect any uncertainty in the reference conditions mean free path, *λ*_0_. We consider this acceptable because a change to *λ*_0_ only affects the calculation of Knudsen number. Any changes can be propagated directly to recomputed values for *α*, *β*, and *γ*. Based onEq. (11), the relative standard uncertainty in *λ* is equal to the relative standard uncertainty of *P*, 0.12 %.

#### 6.1.11 Total Type B Uncertainty in *A* (*Kn*)

From Eqs. (2) and (8), *A* can be expressed in terms of physical variables and constants as:

$$A=\frac{1}{Kn}\left[\frac{QG_{f}3\pi \mu D_{p}}{Ve}-1\right]=\frac{D_{p}}{2\lambda }\left[\frac{QG_{f}3\pi \mu D_{p}}{Ve}-1\right].$$ (20)

As the quantities *λ* and *Q* depend upon pressure, we explicitly express the pressure dependence of *λ* and *Q* from Eqs. (11) and (13) inEq. (20). We also simplify the equation by expressing the first term in the bracket as a product of the codependent quantities *D*_p_ and *P* and a reduced function, *Φ*, involving independent quantities and constants.

$$A=\frac{D_{p}}{2\lambda_{1}P_{0}}[\Phi D_{p}-P]$$ (21)

where

$$\Phi =\frac{3\pi Q_{1}G_{f}\mu P_{\text{cal}}}{Ve}$$ (22)

$$Q_{1}=Q\frac{P}{P_{\text{cal}}}=Q_{\text{cal}}\frac{T_{\text{cal}}}{T}$$ (23)

$$\lambda_{1}=\lambda \frac{P}{P_{0}}=\lambda_{0}{\left(\frac{T}{T_{0}}\right)}^{2}\left(\frac{T_{0}+S}{T+S}\right).$$ (24)

Recalling that the uncertainty contribution from temperature in the mean free path and flow rate has been neglected, the relative standard uncertainty in *A*, *u*_r_(*A*), is computed usingEq. (16) divided by *A*. Combining terms and simplifying the expression, the result is:

$$u_{r}(A)={\left[{\left(\frac{2C-1}{C-1}\right)}^{2}u_{r}{\left(D_{p}\right)}^{2}+{\left(\frac{C}{C-1}\right)}^{2}u_{r}{\left(\Phi \right)}^{2}+{\left(\frac{1}{C-1}\right)}^{2}u_{r}{\left(P\right)}^{2}\right]}^{1/2}$$ (25)

The final uncertainty needed to complete the analysis, is the relative uncertainty in *Φ*. As a simple product, this is computed usingEq. (18). The resulting value of *u*_r_(*Φ*) is 0.46 % for the larger two particle size and 0.70 % for the 20 nm particle size reduced pressure measurements of the slip correction factor. In the limit of large Knudsen number, *C* is large compared to 1, simplifyingEq. (25):

$$u_{r}(A)={\left[2^{2}u_{r}{\left(D_{p}\right)}^{2}+u_{r}{\left(\Phi \right)}^{2}\right]}^{1/2}.$$ (26)

It is seen that the uncertainty in *A* is dominated by twice the uncertainty in the particle diameter, demonstrating how critical the accuracy of the particle size measurement is in determining the slip correction parameter *A*.

#### 6.1.12 Total Uncertainty in 20 nm Diameter

We present, here, the details of the uncertainty calculations for the diameter of the nominal 20 nm particles. The uncertainty analysis is complicated becauseEq. (12) is an implicit equation for *D*_p_. We can simplify the analysis by expressingEq. (12) in the following form, using Eqs. (2) and (22):

$$D_{p}=\frac{CP}{\Phi }=\frac{(1+A2\lambda /D_{p})P}{\Phi }.$$ (27)

In a similar procedure as was used to deriveEq. (16), the differential of *D*_p_ is computed and then divided by *D*_p_ to obtain,

$$\frac{dD_{p}}{D_{p}}=\frac{dP}{P}-\frac{d\Phi }{\Phi }+\frac{P}{\Phi D_{p}}\left(\frac{2Ad\lambda }{D_{p}}+\frac{2\lambda dA}{D_{p}}-\frac{2A\lambda dD_{p}}{D_{p}^{2}}\right).$$ (28)

Making use of the relation between *A* and *C* and using d*λ*/*λ* = –d*P*/*P*,Eq. (28) is recast as

$$\frac{dD_{p}}{D_{p}}=\frac{dP}{P}-\frac{d\Phi }{\Phi }+\frac{C-1}{C}\left(\frac{-dP}{P}+\frac{dA}{A}-\frac{2dD_{p}}{D_{p}}\right).$$ (29)

Solving for d*D*_p_/*D*_p_, one obtains an expression as a function of the independent variables *P*, *A*, and *Φ*.

$$\frac{dD_{p}}{D_{p}}=\frac{C}{2C-1}\left[\frac{C-1}{C}\frac{dA}{A}+\frac{1}{C}\frac{dP}{P}-\frac{d\Phi }{\Phi }\right].$$ (30)

Again, the variance of a linear combination of independent variables is equal to the sum of the variances of the individual variables. The relative uncertainty in *D*_p_, *u*_r_(*D*_p_), is equal to the square root of the variance.

$$u_{r}(D_{p})=\frac{C}{2C-1}{\left[{\left(\frac{C-1}{C}u_{r}(A)\right)}^{2}+{\left(\frac{1}{C}u_{r}(P)\right)}^{2}+{\left(u_{r}(\Phi )\right)}^{2}\right]}^{1/2}.$$ (31)

The value of *D*_p_ from Sec. 4.1.2 is 19.90 nm, the corresponding value of the *Kn* number for the measurement conditions (296.2 K, 98.30 kPa) is 6.973, and the value of *C* based on Table7c is 12.042. The value of *u*_r_(*A*) for the 100 nm particles is computed fromEq. (26) to be 1.169 %. Note that the dominant uncertainty in analyzing the 100 nm data alone was from the Type B uncertainty. Using this information together with the uncertainty values in Table 8, the computed value of *u*_r_(19.90) is 0.61 % for the type B uncertainty.

To obtain the combined uncertainty in the diameter of the nominal 20 nm particles, we need to include repeatability data, which provide a Type *A* uncertainty. The most relevant repeatability data are the variation in the peak voltage for the repeat measurements of the 19.90 nm particles. Ideally we would use the repeat data for ambient pressure, but there are no repeat data for that condition. We use the repeat data for 52.43 kPa, the highest pressure with repeats, and obtain *u*_r_(*V*) = 0.30 %. The change in *D*_p_ corresponding to a change in the peak voltage is *∆ D*_p_ = *C*/(2*C* − 1) *∆ V*/*V*. This results in a Type A uncertainty of 0.15 % for *D*_p_.

The combined relative uncertainty in the 19.90 nm particles is the quadrature sum of the type A and type B uncertainty with a value of 0.63 %.

### 6.2 Nonlinear Fit of Data and Type *A* Uncertainty Analysis

The procedure for determining the constants *α*, *β*, and *γ* in the expression for parameter *A*, seeEq. (3), is to perform a nonlinear least square fit. This is obtained by minimizing the function *S* defined by:

$$S={\sum_{i=1}^{N}\left[A_{i}(\exp)-A_{i}(\mathrm{mod})\right]}^{2}$$ (32)

where *A_i_*(exp) and *A_i_*(mod) are the experimental data points and the calculated model results, respectively. The *DATAPLOT* software package developed at NIST [28] was used for the analysis. A nonlinear least square algorithm published by Press et al. [29] was found to give essentially identical results.

The least square analysis was first carried out for the 26 data points of the 100 nm particles to obtain an expression for the slip correction as a function of *Kn*. This result is needed to compute the diameter of the nominal 20 nm selected by the sizing NDMA. In this case the value of was fixed to equal the Allen et al. [11] value of 1.142 as there were no data from the 100 nm particles at *Kn* less than 1.3. The resulting fit is given by:

A(Kn)=1.142+0.505exp(−0.936/Kn). (33)

**Fig. 7:**
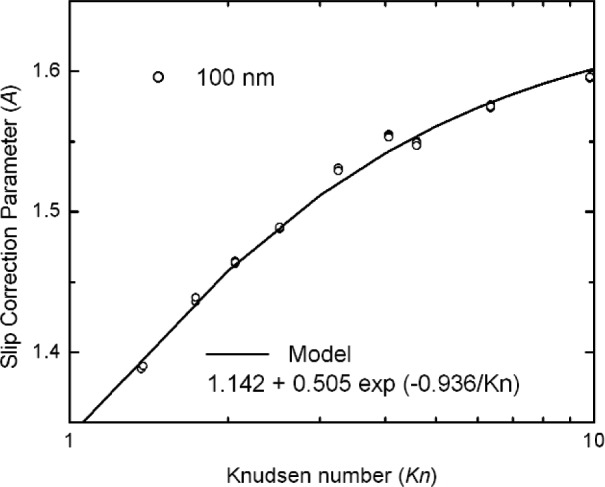
Slip correction parameter *A* from the measurement of 100 nm particles, used in the size calculations for the 20 nm particles.

The comparison of the data and the fit are shown in Fig. 7. This expression for *A*(*Kn*) was then used to compute the diameter of the nominal 20 nm particles leaving the sizing NDMA, as shown in Sec. 4.1.2. Knowing the size allows us to compute *C*(*Kn*) and thus *A*(*Kn*) from the reduced pressure measurements on these particles.

We then carried out a least square analysis for all 56 data points, including the data from all three particle sizes. The resulting expression is given by:

A(Kn)=1.165+0.483exp(−0.997/Kn). (34)

**Fig. 8:**
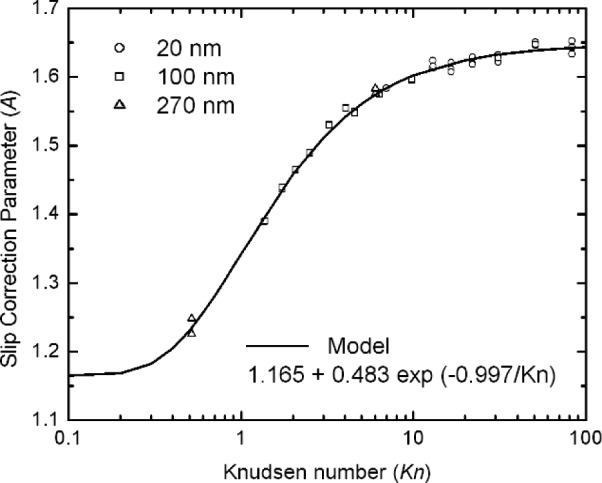
Slip correction parameter *A* from the measurement of 20 nm, 100 nm, and 270 nm particles.

**Fig. 9:**
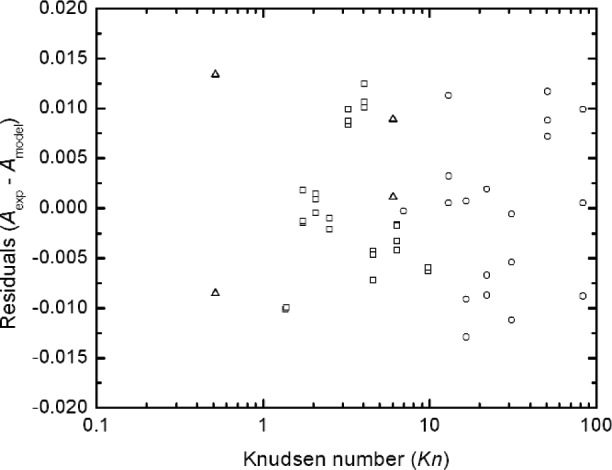
Residuals for slip correction parameter measurements to the fitted model. Circle (19.90 nm), square (100.7 nm), and triangle (269 nm).

The comparison of the data and the model are shown in Fig. 8. A more in depth view of the differences between the data and the model can be seen if we consider a plot of the residuals, (*A*_exp_ – *A*_mod_), as presented in Fig. 9. The residuals are within ±0.015 with the exception of the two points at Knudsen number less than 1. The standard deviation of the residuals is 0.0072. The fact that the residuals are relatively randomly distributed for *Kn* greater than 1 indicates thatEq. (34) accounts for most of the systematic variability of the data. The greater variation for the two data points for the Knudsen number equal 0.515 is a result of the difficulty of generating 270 nm PSL spheres using electrospray. The typical concentration is low, on the order of 1 particle/cm^3^, and this leads to a large uncertainty in the peak voltage. On the other hand, the tight data sets of about 0.005 for the 100 nm particles for a fixed Knudsen number is a result of the high number concentration of about 100 particles/cm^3^ together with the large voltage, which is in the range of 1500 V to 8500 V. The broader data grouping of the residuals of about 0.010 to 0.020 for the 20 nm particles compared to the 100 nm particles at fixed Knudsen number is a result of the lower peak voltage, on the order of 100 V.

The DATAPLOT software also provides the covariance matrix of the parameters, *s* (*p_i_*,*p_j_*), where *p_i_* represents the *i*th parameter, presented in Table 9. This matrix is needed for computing the Type A uncertainty in *A*(*Kn*). The expression *uA*(*A*) for the type A uncertainty of *A*(*Kn*) is given by:

$$u(\alpha +\beta e^{-\gamma /Kn})={\left\{s(\alpha ,\alpha )+{\left(e^{-\gamma /Kn}\right)}^{2}s(\beta ,\beta )+{\left(\frac{\beta }{Kn}e^{-\gamma /Kn}\right)}^{2}s(\gamma ,\gamma )+2(e^{-\gamma /Kn})s(\alpha ,\beta )-2\left(\frac{\beta }{Kn}e^{-\gamma /Kn}\right)s(\alpha ,\gamma )-2\left(\frac{\beta }{Kn}e^{-2\gamma /Kn}\right)s(\beta ,\gamma )\right\}}^{1/2}.$$ (35)

This equation is obtained from a generalization ofEq. (16) and allows for dependent random variables, c.f. Taylor and Kuyatt [30].

The 56 data points were also fitted by the following slightly modified fitting functions:

A=α+(δ−α)⋅exp(−γ/Kn). (36)

The parameter *δ* corresponds to the asymptotic value of *α* + *β* and is expected to be accurately determined from our experiments with much of the data in the large Knudsen number regime. Using Eq. 36, we obtained different parameters and a smaller off-diagonal covariance terms. However, the crucial point is that the computed points based onEq. (36) and the computed points based onEq. (34) agreed within 0.001, which is a factor of 10 smaller than the combined uncertainty. Also, the uncertainty bounds based on the two parameter sets are essentially identical.

### 6.3 Uncertainty Analysis Results for Type A, B, and Combined

**Fig. 10:**
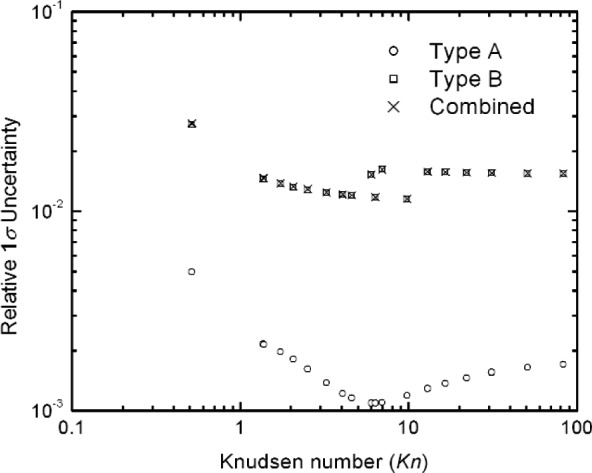
Relative uncertainties of the slip correction parameter *A*.

**Fig. 11:**
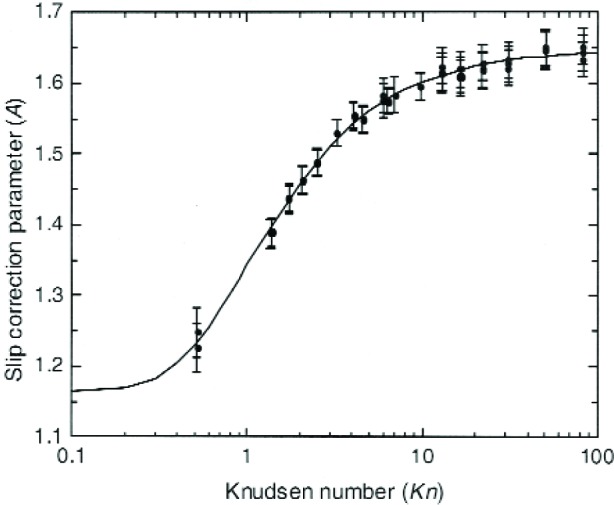
Slip correction parameter *A*, measured and fitted. The bars represent the combined uncertainty.

The results of the Type A uncertainty analysis for *A*(*Kn*) are shown in Fig. 10. It is seen that most of the values are less than 0.2 % and are about a factor of 5 lower than the standard deviation of the residuals. This is because of the large number of measurements (56) and the small number of constants (3). Roughly speaking, one expects the Type A uncertainty to decrease inversely with the square root of the number of measurements. The Type B analysis for *A*(*Kn*), computed usingEq. (25) with the appropriate subsidiary uncertainties, leads to an uncertainty approximately 10 times larger than the Type A uncertainty, as observed in Fig. 10. It is also seen that the combined uncertainty, computed as the quadrature sum of the Type A and Type B components, is essentially identical to the type B uncertainty. Figure 11 presents the best fit curve together with the approximate 1*σ* [(1 × *u*c(*A*)] uncertainty values where *u*c(*A*) is the combined uncertainty (Type A and Type B).

**Fig. 12:**
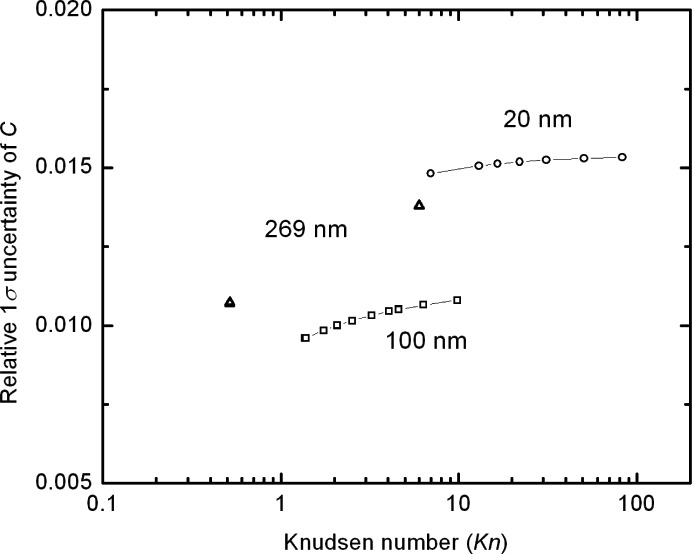
Relative combined uncertainties of the slip correction factor *C*.

While *A*(*Kn*) is the appropriate quantity for finding the best fit, ultimately the quantity of physical interest is the slip correction factor, C(*Kn*), and how its uncertainty varies with the value of the *Kn*. As seen in Fig. 12, the relative uncertainty for *C* is about 1 % for the 100 nm particles, slightly less than 1.5 % for the 20 nm particles, and about 1.2 % for the 270 nm particles.

## 7. Discussion

The nonlinear least square fit of *A*(*Kn*) results in a random (Type A) component of the uncertainty equal or less than 0.2 % for all sizes except for the lowest Knudsen number value. There is a minimum in the Type A uncertainty of about 0.1 % at a *Kn* value of about 7. The combined uncertainty for *A*(*Kn*) based on both the Type A and Type B uncertainties was about 1 % for the 100 nm particles, slightly less than 1.5 % for the 20 nm particles, and about 1.2 % for the 270 nm particles. The Type B uncertainty was approximately an order of magnitude larger than the Type A uncertainty. The dominant contributor to the Type B uncertainty was found to be the uncertainty in the particle size. Other significant contributors to the Type B uncertainty were the geometric factor; the flow rate; and, for the 20 nm particles, the peak voltage. We also note that if the interdependency of the particle diameter and the Knudsen number inEq. (20) were not treated, the computation of Knudsen number depends explicitly on the particle diameter, the uncertainty in *A*(*Kn*) would have been underestimated by about 30 %. Conversely, the dependency of the pressure in the flow measurement and the mean free path resulted in a partial cancellation of the effect of pressure uncertainty. These two situations demonstrate the importance in carefully conducting the uncertainty analysis to prevent over, or more dangerously, underestimation of the uncertainty in calculated results.

Other studies, Allen and Raabe [11] and Hutchins et al. [15], have also measured the slip correction of PSL spheres but have obtained results based on single particle measurements. In both cases, use is made of a nonlinear least square analysis to obtain best fit constants for the slip correction parameter. The major contribution to uncertainty in the fitted parameter appears to be the random component obtained from the least square analysis. While both studies include a listing of the uncertainties in their measurement variables, an assessment of how these uncertainties propagate through to the slip correction parameter is not presented. It is also not clear, from these papers, how one would make use of the stated uncertainties in the constants *α*, *β*, and *γ* when estimating the uncertainty in *A* or in the slip correction factor for a specified value of the Knudsen number as they do not report or comment on the off-diagonal terms in the covariance matrix. In our case, there was a strong correlation between the three parameters which led to a significant contribution from the off-diagonal terms. The last three terms under the square root inEq. (35), arising from the covariances, were approximately 98 % of the diagonal terms at high Knudsen number and, therefore, contributed significantly to the overall Type A uncertainty. Additional study is ongoing to better characterize the uncertainty in these studies [11, 15] and the Millikan data [2] reanalyzed by Allen and Raabe [10].

**Fig. 13:**
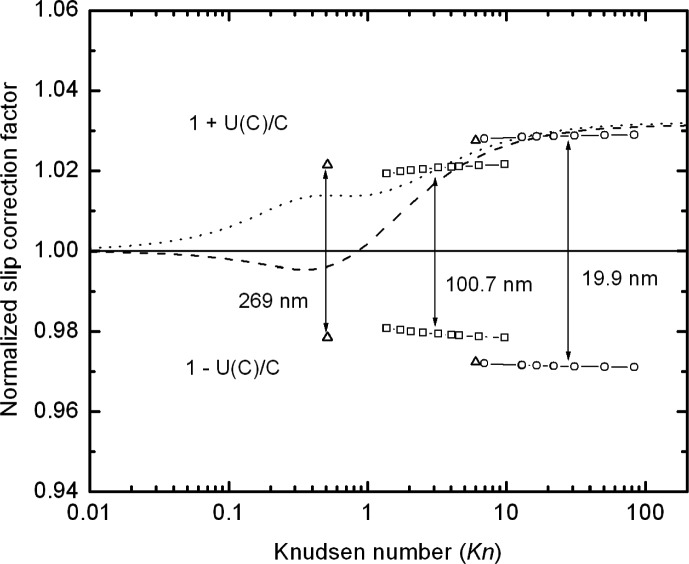
Comparison of the slip correction factors for the reported PSL particle cases with our measurement result. The dashed line is for Allen and Raabe (1985), the dotted line is for Hutchins et al. (1995), and the solid line is for the current study. Confidence intervals for the expanded (2 sigma) uncertainty, *U*(*C*), are given for our data.

The best fit results of Allen and Raabe [11] and Hutchins et al. [15] are compared to our results in Fig. 13. It is seen that the agreement is better with Allen and Raabe’s result for smaller Knudsen numbers, but that the deviation increases with increasing Knudsen number. The asymptotic value of *A* (*Kn*) for large *Kn* is 1.648 for our study compared to a value of 1.70 for the other two studies. The value of 1.70 is slightly outside the 95 % confidence interval for our result, 1.596 – 1.699. The fact that our result is smaller than the other results may be because of the much smaller particle size in our study, 20 nm compared to about 1000 nm or larger for the other studies, and may be an indication that the slip correction parameter depends on more than the Knudsen number as the particle size decreases to the nanometer size range.

**Fig. 14:**
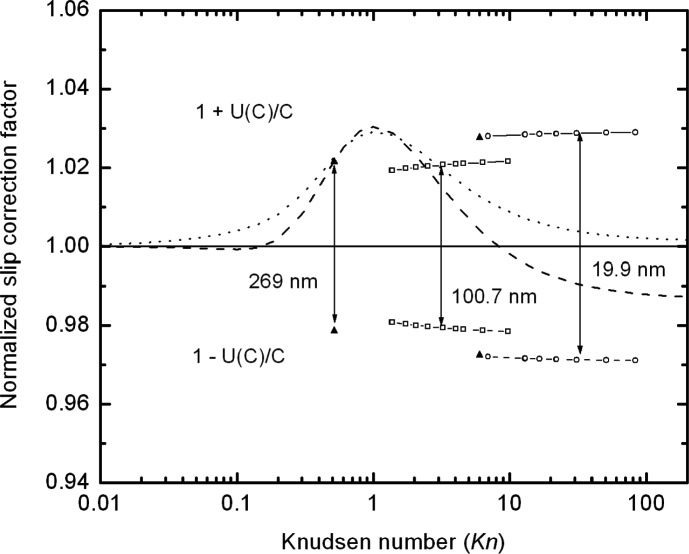
Comparison of the slip correction factors for the reported oil drop cases with our measurement result. The dashed line is for Allen and Raabe (1982), the dotted line is for Rader (1990), and the solid line for the current study. Confidence intervals for the expanded (2 sigma) uncertainty, *U*(*C*), are given for our data.

Figure 14 shows a comparison of our results with those obtained for oil drops in the Millikan cell [10,12]. The largest differences between our PSL results and the oil drop results are near a Knudsen number of one where the oil drop results are as much as 3 % higher than our PSL results. In this case, the difference in the particle surface as well as the difference in particle size at comparable Knudsen numbers is likely contributing to the difference in the measured results. Still, it is noteworthy that the results agree within 3 % over the experimental range of the Knudsen number.

In the present study, electrospray was used to generate the PSL particles. This generation method produces smaller droplets and, thus, leads to less surface contamination from the suspending water. Another significant effect of electrospray is the very low production of multiplets compared to pneumatic atomization. The high field produced in the electrospray may be leading to the breakup of doublets in the droplets. Without electrospray, it would be very difficult to generate a 20 nm PSL aerosol from a liquid suspension. Previous measurements of an aerosol produced by a pneumatic nebulization of 25 nm spheres indicated a peak particle size corresponding to a multiplet of the primary spheres [31].

One limitation of using differential mobility analysis for determining the slip correction is the need for an independent measurement of the particle diameter. The results for *A* are very sensitive to the particle diameter. A 1 % change in the diameter results in a 2 % change in the value of *A*. There is a need for an independent accurate measurement method for particles in the size range between 2 nm and 100 nm to obtain more accurate slip correction data.

An additional statement must be made regarding the size calibration of the 20 nm particles. The size was initially determined using slip correction parameter results from measurements conducted on the 100 nm particles. This diameter was then used to obtain results across a greater range of *Kn*. The inclusion of the additional data points had the potential to shift the fitted slip correction parameter near the Knudsen number used to calculate the diameter of the 20 nm particles. A consistency calculation was, therefore, required and conducted to check the results. Using the fit of the slip correction parameter from the entire set of data resulted in only about a 0.003 % change in the diameter, thus establishing consistency.

## 8. Conclusions

1. Electrical mobility analysis with a condensation particle counter was successfully used to measure the slip correction factor for nanometer-sized particles as a function of system pressure, for pressures as low as 8.27 kPa.
2. Based on the nonlinear least square fit for the slip correction parameter *A*(*Kn*) of the Knudsen and Weber form, the asymptotic value of (*α* + *β*) for the free molecular regime is 1.648. This is about 3 % smaller than two previous PSL slip correction results.
3. The dominant uncertainty contribution to the slip correction parameter is from the particle diameter. Other significant contributors are the geometric constant and the voltage. Proper treatment of interdependencies in both Type A and Type B uncertainties is crucial to obtaining accurate uncertainty limits.
4. Over the Knudsen range from 0.5 to 83, our results for the slip correction factor *A*(*Kn*), measured for particles as small as 20 nm, is within about 3 % of the values obtained by other researchers [11, 15] who have used PSL spheres with typical diameters of 1000 nm or larger. The largest difference is observed at large Knudsen numbers.
5. Comparison of our results for the slip correction factor *A*(*Kn*) is within 3 % of the values obtained from oil drop studies, with the maximum difference occurring at Knudsen numbers of around 1.

## Figures and Tables

**Table 1:** Previous slip correction parameters with the Knudsen and Weber form to correct for air. All parameters were modified for the mean free path of 67.3 nm at 101.3 kPa and 23 °C

Author (year)	Material	*α + β*	*α*	*β*	*γ*
Knudsen and Weber (1911) a	Glass spheres	1.570	1.034	0.536	1.219
Millikan (1923) b	Oil drops	1.615	1.209	0.406	0.893
Allen and Raabe (1982)	Oil drops	1.626	1.155	0.471	0.596
Allen and Raabe (1985)	PSL spheres	1.700	1.142	0.558	0.999
Rader (1990) c	Oil drops	1.650	1.209	0.441	0.779
Hutchins et al. (1995)	PSL spheres	1.700	1.231	0.469	1.178

a Knudsen and Weber [3] originally reported the slip correction parameter *A* as (0.683 + 0.354exp (−1.845 / *Kn*)) using the mean free path of 100.65 nm at 101.3 kPa and 20.2 °C.

b 94.17 nm was originally used for the mean free path at standard conditions.

c 67.4 nm was originally used for the mean free path at standard conditions.

**Table 2:** Kinetic theory *ϕ* values for the viscosity formula [21]

Model	*ϕ*
Maxwell	0.33
Boltzmann	0.3502
Chapman and Enskog^a^	0.499^b^
	0.491^c^

a The same theory was developed independently, hence it is called the Chapman and Enskog model.

b For hard elaastic spheres with not repulsive force between the molecules.

c For hard elastic spheres with a repulsive force between the molecules

**Table 3:** Physical constants for the electrical mobility equation at 23 °C and 101.3 kPa

Constant	Symbol	Value	Reference
Electronic charge	*e*	1.602176 × 10^−19^ kg m^2^ s^−2^ V^−1^	NIST online data base [23]
Mean free path of air	*λ*	67.30 nm	Allen and Raabe [10]
Viscosity of air	*µ*	1.83245 × 10^−5^ kg m^−1^ s^−1^	Birge [19]

**Table 4:** Diameter and uncertainty for the particles used in this study

Material	Mean diameter (nm)	Relative standard uncertainty
SRM 1963	100.7	0.50 %
SRM 1691	269.0	0.68 %
Duke 3020A	19.90^a^	0.63 %

a Size selected using a Nano differential mobility analyzer

**Table 5:** Repeatability test of peak voltage of SRM 1963 particles for two different days. The sheath air of 5.918 L/min (98.8 cm^3^/s) flow was used

		11/14/2003	10/30/2003
NDMA	Measurement		
		98.8 kPa & 22.3 °C	98.0 kPa & 22.5 °C
1	1	8353.4	8305.2
	2	8359.1	8296.3
	3	8349.2	8279.8
	4	8352.9	8310.9
	5	8350.7	8303.5
2	6	8354.0	
	7	8357.6	
	8	8353.5	
	9	8358.0	
	10	8350.2	

Average		8353.8 ±3.2	8302.7 ±5.2
	Average Value:		
Value:		(8510.0)^a^	(8509.8)^a^

a Voltage adjusted for a pressure of 101.32 kPa and a temperature of 23 °C

**Table 6:** Comparison of the electrical breakdown voltage at reduced pressure conditions with the peak voltage measurements

Pressure (kPa)^a^	Breakdown voltage in air (*V*)	Peak voltage of SRM 1963^b^ (*V*)
1.13	650	
1.85	1170	
2.43	1440	
3.41	1830	
4.08	2080	
5.39	2550	
6.87	3050	695
10.08	3880	1075
19.29	5550	1900
28.58	7100	2850

a 1 mmHg = 133.32 Pa

b Peak voltage was measured with a sheath air flow of 100 cm^3^/s.

**Table 7a:** Measurements with the 100 nm particles for the slip correction factor

		Sheath/Aerosol				
*P* (kPa)^a^	*T* (K)	*Q* (L/min)	Peak *V*^b^	*Kn*	*C*	*A*
98.80	295.5	5.918/ 0.60	8470.0	1.367	2.897	1.388
98.00	295.7	5.918/ 0.60	8418.2	1.379	2.915	1.389
78.06	296.4	3.288/ 0.17	7040.5	1.738	3.495	1.436
78.05	296.4	3.289/ 0.17	7038.7	1.738	3.496	1.436
78.06	296.5	3.289/ 0.17	7028.2	1.739	3.502	1.439
65.81	297.2	4.477/ 0.20	6118.9	2.068	4.028	1.464
65.81	297.3	4.478/ 0.20	6119.9	2.066	4.028	1.466
65.82	297.2	4.476/ 0.20	6125.9	2.066	4.024	1.464
54.18	297.5	5.725/ 0.24	5200.2	2.515	4.744	1.488
54.19	297.5	5.724/ 0.24	5204.0	2.514	4.741	1.488
54.19	297.5	5.724/ 0.24	5203.9	2.514	4.741	1.488
41.88	297.5	7.820/ 0.40	4125.6	3.252	5.978	1.531
41.88	297.5	7.820/ 0.40	4129.2	3.252	5.973	1.529
41.88	297.6	7.820/ 0.40	4127.8	3.254	5.976	1.529
33.53	297.5	9.913/ 0.45	3370.1	4.063	7.320	1.555
33.53	297.5	9.913/ 0.45	3373.5	4.063	7.313	1.554
33.52	297.5	9.916/ 0.45	3373.2	4.065	7.314	1.553
29.60	297.2	4.371/ 0.34	3039.1	4.596	8.109	1.547
29.61	297.3	4.370/ 0.34	3034.9	4.595	8.123	1.550
29.61	297.3	4.370/ 0.34	3035.7	4.595	8.120	1.550
21.41	297.4	6.099/ 0.43	2236.7	6.358	11.024	1.577
21.41	297.4	6.099/ 0.43	2240.0	6.358	11.008	1.574
21.41	297.4	6.101/ 0.43	2238.8	6.360	11.016	1.575
13.87	297.5	9.504/ 0.58	1479.4	9.822	16.672	1.596
13.87	297.5	9.504/ 0.58	1479.1	9.822	16.674	1.596
13.88	297.6	9.504/ 0.58	1480.4	9.817	16.665	1.596

a 1 mmHg = 133.32 Pa.

b Converted for the sheath flow rate of 6 L/min (100 cm^3^/s).

**Table 7b:** Measurements from the doubly charged 270 nm particles for the slip correction factor

		Sheath/Aerosol				
*P* (kPa)^a^	*T* (K)	*Q* (L/min)	Peak *V*	*Kn*	*C*	*A*
98.50	296.2	1.976/ 0.20	6629.0	0.515	1.631	1.226
98.50	296.3	1.976/ 0.20	6584.0	0.515	1.642	1.247
84.26	296.1	3.566/ 0.62	1861.9	6.014	10.476	1.436
84.26	296.2	3.567/ 0.62	1853.9	6.014	10.521	1.580

a 1 mmHg = 133.32 Pa.

**Table 7c:** Measurements from the 20 nm particles for the slip correction factor

		Sheath/Aerosol				
*P* (kPa)^a^	*T* (K)	*Q* (L/min)	Peak *V*^b^	*Kn*	*C*	*A*
98.30	296.2	5.918/ 0.60	403.5	6.973	12.042	1.584
52.43	294.7	2.172/ 0.34	219.1	12.984	22.091	1.624
52.43	295.0	2.175/ 0.34	220.4	13.001	21.972	1.613
52.45	295.3	2.174/ 0.34	220.0	12.998	22.017	1.617
41.25	295.7	2.895 0.47	174.1	16.576	27.870	1.621
41.28	295.8	2.894 0.47	175.2	16.573	27.702	1.611
41.28	295.8	2.894 0.47	175.6	16.573	27.637	1.607
31.06	296.1	3.893 0.60	132.4	22.049	36.686	1.619
31.08	296.1	3.891 0.60	132.3	22.040	36.710	1.620
31.08	296.2	3.892 0.60	131.6	22.049	36.901	1.628
22.02	296.2	5.627/ 0.71	94.4	31.112	51.483	1.623
22.02	296.2	5.627/ 0.71	93.8	31.112	51.775	1.632
22.01	296.3	5.633/ 0.71	94.0	31.144	51.726	1.629
13.48	296.3	9.386/ 0.97	57.2	50.859	84.937	1.650
13.48	296.3	9.386/ 0.97	57.3	50.859	84.842	1.649
13.49	296.4	9.379/ 0.97	57.4	50.831	84.612	1.645
8.27	296.3	15.512/ 1.38	35.6	82.934	136.588	1.635
8.27	296.3	15.512/ 1.38	35.4	82.934	137.305	1.644
8.27	296.3	15.512/ 1.38	35.2	82.934	138.059	1.653

a 1 mmHg = 133.32 Pa.

b Converted for the sheath flow rate of 6 L/min (100 cm^3^/s).

**Table 8:** Summary of uncertainties that contribute to the slip correction parameter uncertainty and to the 19.90 nm diameter uncertainty

Variable	Value	% uncertainty
e, electronic charge	1.6022 E-19 kg m^2^ s^−1^ V^−1^	negligible
*D*_p_, particle diameter		
Selected with NDMA from Duke 3020A	19.90 nm	0.63 %
SRM 1963	100.7 nm	0.50 %
SRM 1691	269 nm	0.68 %
*P*, reduced pressure	8 kPa to 100 kPa	0.12 %
*P*_cal_, flow calibration pressure	100 kPa, nominal	0.12 %
*P_0_*, reference pressure for λ	101.33 kPa	Fixed
*T*, temperature	296.15 (nominal)	0.03 %^a^
*Q*_1_, reduced flowrate	6 L/min	0.06 %
*V*, peak voltage		
SRM® 1963 and SRM® 1691	1500 V to 8500 V	0.21 %
19.90 nm	100 V to 400 V	0.56 %
*µ*, viscosity of air	1.8325 E-5 kg m^−1^ s^−1^	0.09 %
*G*_f_ = ln(*r*_1_/*r*_2_)/2*πL*, geometric constant	2.264 m^−1^	0.38 %
λ_1_, reduced mean free path	673 nm at 296.15 °K	negligible^b^
*ΔL*, settling distance	negligible	negligble
*Φ*=3*πµQ*_1_*G*_f_*P*_cal_/ *V*e		
SRM® 1963 and SRM® 1691		0.46 %
19.90 nm		0.70 %
19.90 nm for set voltage^c^		0.48 %

a The temperature uncertainty has a negligible contribution to the uncertainty in *A (Kn).*

b The only uncertainty arises from the temperature uncertainty which is negligible.

c The contribution of the voltage uncertainty to *Φ* is 0.24 % when the voltage is set at a 398 V for generating 19.90 nm PSL spheres.

**Table 9:** Estimated Variance-Covariance matrix components and correlation coefficients, computed as $\rho (x,y)/\sqrt{s(x,x)s(y,y)}$ for the nonlinear least square fit of the slip correction parameter. Correlation coefficients near one denote strong correlation; near negative one, strongly anti-correlation. Correlation coefficients near zero signify independence

Quantity	Value
*s(α, α)*	1.2119 × 10^−4^
*s*(*β, β)*	1.1009 × 10^−4^
*s(γ, γ*)	1.5604 × 10^−3^
*s*(*α*, *β*)	−1.1400 ×10^−4^
*s*(*α*, *γ)*	4.0103 × 10^−4^
*s*(*β, γ*)	−3.5989 × 10^−4^
*ρ (α, β)*	−0.987
*ρ (α, γ)*	0.922
*ρ (β, γ)*	−0.868

## Footnotes

1 Certain commercial equipment, instruments, or materials are identified in this paper to foster understanding. Such identification does not imply recommendation or endorsement by the National Institute of Standards and Technology, nor does it imply that the materials or equipment identified are necessarily the best available for the purpose.
